# Mechanisms of Heat-Induced Sleep Disruption in Aging: A Narrative Review

**DOI:** 10.3390/clockssleep8030043

**Published:** 2026-07-08

**Authors:** Neriman Ezgin, Jelena Krčum, Nikola Šutulović, Maja Pavlović, Emilija Djurić, Dušan Mladenović, Milena Vesković, Arif E. Cetin, Aleksandra Rašić-Marković, Olivera Stanojlović, Dragan Hrnčić

**Affiliations:** 1 Institute of Medical Physiology “Richard Burian”, Faculty of Medicine, University of Belgrade, 11000 Belgrade, Serbia; 2 Department of Biotechnology, Institute of Natural and Applied Sciences, Cukurova University, Adana 01330, Turkey; 3 Faculty of Medicine, University of Belgrade, 11000 Belgrade, Serbia; 4 Faculty of Sciences and Mathematics, University of Priština in Kosovska Mitrovica, Lole Ribara 29, 38220 Kosovska Mitrovica, Serbia; 5 Institute of Pathophysiology “Ljubodrag Buba Mihailovic”, Faculty of Medicine, University of Belgrade, 11000 Belgrade, Serbia; 6 Izmir Biomedicine and Genome Center, Balcova, Izmir 35340, Turkey; 7 Izmir International Biomedicine and Genome Institute, Dokuz Eylul University, Balcova, Izmir 35340, Turkey; 8 Department of Electrical and Electronics Engineering, Istanbul Atlas University, Kagithane, Istanbul 34408, Turkey; 9 Department of Biophysics, Faculty of Medicine, Dokuz Eylul University, Izmir 35340, Turkey

**Keywords:** heat, sleep, heatwaves, thermoregulation, cooling, aging

## Abstract

Rising global temperatures and more frequent heat waves pose a growing threat to public health, particularly among older adults. Age-related declines in thermoregulatory capacity such as reduced sweating, impaired vasodilation, and diminished hypothalamic responsiveness make it more difficult to cope with elevated nighttime temperatures. These thermal challenges disrupt sleep by prolonging sleep onset, fragmenting slow-wave and REM sleep, and suppressing melatonin secretion. Importantly, sex-related differences in thermoregulatory aging—particularly menopause-associated vasomotor instability in women and testosterone-related autonomic changes in men—further modulate individual vulnerability to heat-induced sleep disruption. Beyond sleep, heat-induced stress affects metabolic, cardiovascular, and immune systems, promoting insulin resistance, endothelial dysfunction, sympathetic overactivation, and chronic inflammation. Socioeconomic disparities, urban heat island exposure, and differential access to cooling infrastructure function as critical environmental modifiers that amplify biological vulnerability, particularly in disadvantaged older populations. This narrative review aims to synthesize current knowledge on the specific thermoregulatory mechanisms of sleep disruption induced by heat stress in older adults. Understanding these interactions is crucial for developing effective interventions, including environmental cooling, circadian-aligned behaviors, and targeted public health strategies, to mitigate the compounded risks of heat exposure and preserve healthy sleep in aging populations. However, many proposed mechanistic pathways are primarily derived from animal models, and controlled human studies specifically targeting heat-exposed older adults remain scarce.

## 1. Introduction

Climate change is increasing the frequency and intensity of heat waves across the world, making extreme heat one of the most pressing environmental health challenges of our time. While heat exposure is often associated with acute outcomes such as heat stroke or cardiovascular mortality, its broader effects on vital physiological processes are becoming increasingly recognized. One such process is sleep, which plays a critical role in maintaining physical and mental health. In recent years, growing evidence has suggested that elevated nighttime temperatures can significantly disrupt sleep, particularly among older adults [1].

Sleep is a fundamental biological process that supports cognitive functioning, emotional regulation, immune responses, and metabolic balance. Under physiological conditions, the human body prepares for sleep through a gradual decline in core body temperature during the evening. This thermoregulatory process helps initiate and maintain sleep. However, when nighttime temperatures remain high, the body’s ability to dissipate heat is reduced. As a result, individuals may experience difficulty falling asleep, more frequent awakenings during the night, and shorter overall sleep duration [2,3].

Very recent studies have highlighted the association between ambient temperature and sleep outcomes. For instance, Li et al. (2025) [3], in a large-scale repeated-measures epidemiological study of over 23 million sleep records, reported that each 10 °C increase in ambient temperature was associated with a 20.1% higher likelihood of insufficient sleep and an average reduction of nearly ten minutes in sleep duration [3]. Similarly, Zhou et al. (2024), in a population-based observational study of older adults, found that exposure to heatwaves was associated with significantly reduced total sleep time [4]. These findings suggest a robust relationship between environmental heat exposure and sleep duration at the population level, although causal mechanisms cannot be directly inferred from these observational designs.

Controlled experimental and physiological studies further support these associations, indicating that exposure to elevated ambient temperatures is linked to reduced sleep efficiency, decreased REM sleep, and increased nocturnal wakefulness [5]. Evidence from systematic reviews also consistently reports that ambient heat is associated with poorer sleep quality across diverse populations and climatic conditions [6].

The older adults have been recognized as particularly vulnerable to heat-induced sleep disturbances [3]. Namely, aging is associated with a decline in thermoregulatory capacity, including reduced skin blood flow and a diminished sweating response, which makes it more difficult to regulate body temperature in hot environments [7,8]. Disruptions in thermal balance can directly affect sleep architecture and quality since sleep and thermoregulation are closely linked through neural pathways in the preoptic anterior hypothalamus (POA) [9]. However, these mechanisms are much more complex in older adults and still not comprehensively summarized.

Understanding how heat exposure affects sleep in the elderly population has become an important public health concern due to climate changes and increased number of extreme heat events worldwide. Therefore, this narrative review aims to synthesize current knowledge on the specific thermoregulatory mechanisms of sleep disruption induced by heat stress in older adults.

Specifically, we address the following questions: (i) How do aging-related thermoregulatory changes increase vulnerability to heat-induced sleep disruption? (ii) What molecular and neurobiological mechanisms mediate this relationship? (iii) What intervention strategies may mitigate these risks in older populations?

Accordingly, this narrative review synthesizes current evidence on heat-related sleep disruption in older adults, with a primary focus on thermoregulatory vulnerability, underlying neurobiological and molecular mechanisms, and secondary on potential intervention strategies aimed at mitigating heat-associated sleep disturbances in aging populations.

## 2. Methods and Search Strategies

A literature search for this narrative review was performed using PubMed, Scopus, and Web of Science databases to identify relevant English-language studies published in period 1995–March 2026.

Search terms included combinations of keywords such as “heat stress,” “heatwaves,” “sleep,” “aging,” “older adults,” “thermoregulation,” “circadian rhythm,” “preoptic area,” “glymphatic system,” “AQP4,” “oxidative stress,” “melatonin,” “sleep fragmentation,” and “climate change.” Additional relevant studies were identified through manual screening of reference lists from key review articles and primary research papers.

Priority was given to human epidemiological studies, clinical sleep research, and systematic reviews examining associations between ambient heat exposure and sleep outcomes, particularly in older adults. Experimental and mechanistic animal studies were also included when they provided relevant insights into thermoregulatory, neurobiological, circadian, metabolic, or molecular pathways underlying heat-related sleep disruption.

The included literature spans approximately the past three decades, allowing coverage of both foundational mechanistic studies and recent large-scale observational and wearable-based evidence.

The review focused on four main thematic domains: (i) aging-related changes in thermoregulation and sleep physiology, (ii) effects of environmental heat exposure on sleep, (iii) neurobiological and molecular mechanisms linking heat stress and sleep disruption, and (iv) public health and adaptation strategies.

Exclusion criteria were: non-peer-reviewed sources, articles written in languages other than English, conference abstracts, and studies not directly related to the aims of this review.

As this is a narrative review, no formal systematic review methodology (e.g., PRISMA framework, quantitative synthesis, or risk-of-bias assessment) was applied. The aim was to provide an integrative and conceptually structured synthesis of the literature rather than a quantitative evidence aggregation.

## 3. Sleep and Aging

Sleep is a biologically essential process that supports neurocognitive performance, metabolic regulation, immune competence, and cardiovascular stability. Although sleep architecture and its systemic effects are important throughout the lifespan, aging is associated with distinct alterations in sleep duration, depth, and neuroendocrine coordination. These changes can compromise key restorative functions, including memory consolidation, glymphatic clearance of neurotoxic proteins such as β-amyloid and tau largely demonstrated in animal and experimental models. Consequently, aging-related sleep alterations increase susceptibility to metabolic, immunological, and neurodegenerative disturbances, making sleep a critical determinant of health in later life.

The older adults commonly experience reductions in slow-wave and REM sleep, increased sleep fragmentation, prolonged sleep latency, and decreased melatonin secretion [10,11,12]. These ageing-related alterations in sleep quantity and quality could contribute to cognitive decline, as well as some other adverse events. The reduction in slow-wave sleep is particularly important because this stage is closely associated with growth hormone secretion and synaptic homeostasis. Impairment of these processes has been implicated in the development of neurodegenerative diseases, including Alzheimer’s disease [13].

Aging-related alterations in neurotransmitter systems further destabilize sleep architecture. Declines in GABAergic and serotonergic signaling reduce cortical inhibition and disrupt circadian regulation, contributing to fragmented sleep and circadian misalignment. At the same time, dopaminergic and noradrenergic modulation of REM sleep primarily supported by experimental and animal studies may impair emotional regulation and cognitive processing, increasing susceptibility to mood disorders and executive dysfunction [14]. These neurobiological changes also have systemic consequences. Given these aging-related changes in sleep architecture and physiological resilience, older adults may be particularly sensitive to environmental stressors.

## 4. Mechanisms of Thermoregulation

Maintaining body temperature is a fundamental component of homeostasis in mammals. The hypothalamic preoptic area (POA) acts as the primary regulatory center of thermoregulation by integrating signals from peripheral thermoreceptors located in the skin and visceral organs with internal temperature information from central sensors. Through this integration, the POA coordinates autonomic, endocrine, and behavioral responses that allow the body to adapt to both environmental and internal thermal challenges as primarily demonstrated in experimental neurophysiology and animal studies [9,15]. Thermal information from the external environment is initially detected by peripheral thermoreceptors located in the skin, mucous membranes, and deep body tissues. From there, thermosensory pathways project through brainstem nuclei such as the lateral parabrachial nucleus before reaching the preoptic area of the hypothalamus, where peripheral and central thermal signals are integrated to coordinate appropriate thermoregulatory responses based mainly on animal and translational experimental models [16,17].

The POA integrates these peripheral signals with central temperature information and activates appropriate efferent responses. During heat exposure, this may include sympathetic-mediated vasodilation, sweating, and behavioral adaptations such as seeking cooler environments. In contrast, cold exposure triggers shivering, brown adipose tissue activation, and peripheral vasoconstriction to conserve heat as described primarily in physiological and experimental studies [15].

This peripheral–central integration allows the body to regulate temperature efficiently while also interacting with endocrine and immune systems. Hormonal signals such as thyroid hormones and other metabolic mediators can modify thermoregulatory responses during stress, because thyroid hormones regulate metabolic heat production and energy balance and interact with neural thermoregulatory circuits and immune pathways based largely on experimental and physiological evidence rather than direct causal human interventional data [18]. The interaction between peripheral and central thermoregulatory systems is summarized in Table 1.

Within POA, warm-sensitive neurons respond to small fluctuations in temperature and regulate multiple thermoregulatory processes. Through sympathetic and parasympathetic pathways, these neurons control physiological responses such as sweating, cutaneous vasodilation, shivering, and thermogenesis mediated by brown adipose tissue as demonstrated in review-based and experimental physiological evidence [19,20]. When environmental temperatures rise, increased activity in POA neurons promotes heat-loss mechanisms, including vasodilation and sweating. In contrast, exposure to cold activates posterior hypothalamic circuits that stimulate heat-producing responses. Importantly, thermoregulation is closely linked with sleep physiology. The reduction in core body temperature that typically precedes sleep onset is largely mediated by POA neuronal activity, highlighting the tight coupling between circadian rhythms, thermoregulation, and sleep regulation supported by human studies with experimental validation [20,21].

The thermosensitive POA neurons relays on Transient Receptor Potential (TRP) channels, which play an essential role in detecting temperature changes and initiating appropriate thermoregulatory responses. Among these channels, TRPM2 has been shown to function as a heat-sensitive ion channel in POA neurons, responding to increased brain temperatures and influencing body temperature regulation, and TRPV1, a classic heat-activated TRP channel, is implicated in thermal detection and warm sensing in the nervous system based on mechanistic animal and molecular-level studies [22,23]. TRPM2 is highly expressed in heat-sensitive neurons of the preoptic area and contributes to the activation of heat-dissipation mechanisms. Experimental studies have shown that TRPM2 promotes peripheral heat loss and reduces body temperature, whereas dysfunction or deletion of this channel can impair vasodilation and behavioral cooling responses, potentially leading to hyperthermia as demonstrated in animal experimental models [24,25].

Another thermosensitive channel, TRPV1, is involved in detecting high temperatures and has been associated with thermoregulatory instability in certain physiological conditions. Fluctuation in estrogen levels can influence TRPV1 expression and thermal sensitivity, which may contribute to symptoms such as menopausal hot flashes as shown in animal experimental studies [26]. Thermoregulatory responses are further shaped by interactions between inhibitory GABAergic neurons and excitatory glutamatergic neurons within the POA. GABAergic neurons suppress thermogenesis during heat exposure and play a key role in promoting non-rapid eye movement (NREM) sleep by inhibiting wake-promoting brain regions, whereas glutamatergic neurons can modulate both heat production and heat-loss pathways, as well as destabilize NREM sleep and suppress REM sleep depending on environmental and physiological conditions as demonstrated in experimental neurocircuit and animal studies [9,27]. The coordinated activity of these neuronal populations contributes to the nocturnal decline in core body temperature that facilitates sleep onset.

The major molecular and neuronal components involved in central thermoregulation are summarized in Table 2.

### 4.1. Thermoregulation and Circadian Rhythm

Thermoregulation is not an isolated physiological process but is closely integrated with circadian rhythms and behavioral regulation. Core body temperature exhibits robust daily fluctuations driven by the central circadian clock in the suprachiasmatic nucleus (SCN), which are synchronized with sleep–wake cycles as demonstrated primarily in human physiological and circadian rhythm studies as well as experimental animal models [28,29]. Thermosensitive neurons in the POA receive circadian input from the SCN and other hypothalamic circuits, helping to coordinate the nocturnal decline in core body temperature with sleep onset, based largely on experimental animal and translational neuroscience studies [30,31]. Moreover, transitions between sleep and wake states produce rapid changes in brain and core body temperature, reflecting tight coupling between thermoregulatory circuits, circadian timing, and behavioral state regulation, as shown in both human physiological recordings and experimental models [30].

Chronic heat exposure may also influence neural circuits associated with emotional regulation and arousal. Changes in projections between the POA and regions such as the posterior paraventricular thalamus have been linked to increased stress responses and enhanced arousal, both of which can further disrupt sleep continuity, as primarily demonstrated in experimental animal models and preclinical neuroscience studies [32]. These findings suggest that thermoregulation is closely intertwined with both physiological and psychological processes affecting sleep.

### 4.2. Changes in Thermoregulation with Aging

**Figure 1:**
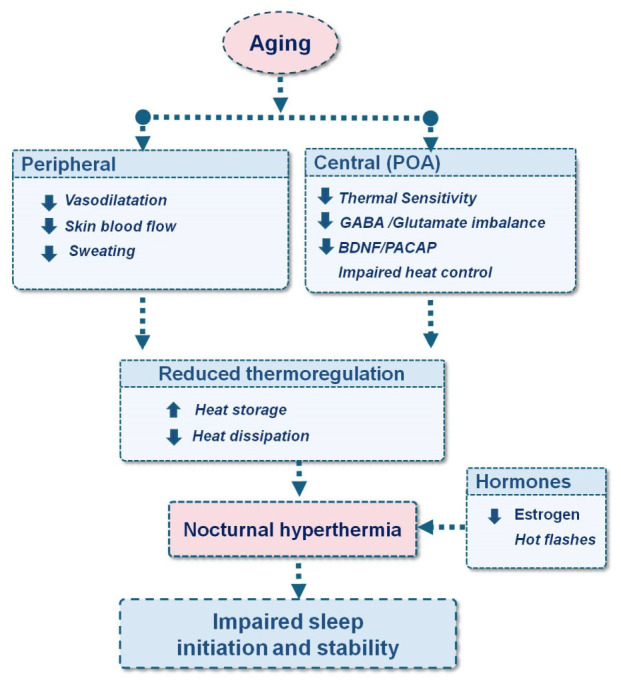
Aging-related disruption of thermoregulation and sleep.

Thermoregulatory efficiency declines with aging due to reduced sensitivity of hypo-thalamic POA neurons and impairments in peripheral heat-dissipation mechanisms, including sweating and vasodilation, as demonstrated primarily in systematic reviews and human studies [33,34]. Aging-related alterations may disrupt integrated thermoeffector loops primarily driven by core temperature feedback and reduce overall thermoregulatory precision, as summarized in review-based evidence integrating human and experimental data [35]. Studies indicate that maximal cutaneous blood flow is significantly lower in older adults than in younger individuals, and attenuated sudomotor and vasodilatory responses further compromise heat loss, as shown in human experimental and clinical studies [36]. Namely, cutaneous vascular regulation depends on dual sympathetic pathways, including a tonic noradrenergic vasoconstrictor system and an active cholinergic vasodilator system activated during heat stress, involving mediators such as nitric oxide, vasoactive intestinal peptide, prostaglandins, and substance P; these mechanisms are primarily characterized in human studies and translational experimental research [37,38]. This decline diminishes the ability of older adults to maintain core temperature during heat exposure, increasing the risk of nocturnal hyperthermia, sleep fragmentation, and heat-related cardiovascular complications (as illustrated in Figure 1) [7,15]. In parallel, older individuals exhibit increased dry heat gain and greater cumulative heat storage under both dry and humid conditions, indicating elevated thermal strain despite relatively preserved evaporative heat loss, as demonstrated in human experimental thermoregulation studies [39]. Aging-related reductions in heat dissipation can disrupt nocturnal cooling and sleep architecture, increasing fragmentation.

At the neuronal level, aging-related changes in the balance of inhibitory GABAergic and excitatory glutamatergic signaling within the POA influence both thermoregulation and sleep. GABAergic neurons promote sleep initiation and suppress heat-generating circuits, whereas glutamatergic neurons modulate heat production and heat loss as well as arousal states, as demonstrated primarily in experimental animal models and neurophysiological circuit studies. GABAergic sleep-promoting networks within the hypothalamus, particularly the ventrolateral preoptic area (VLPO), inhibit wake- and arousal-related hypothalamic regions, including dorsomedial hypothalamic (DMH) pathways that contribute to sympathetic activation and thermogenesis. Conversely, activation of DMH circuits promotes sympathetic outflow, thermogenic responses, and wake-associated autonomic arousal, based mainly on experimental animal and circuit-level studies [40]. In addition, molecular pathways such as brain-derived neurotrophic factor (BDNF) affect POA thermal sensitivity and synaptic plasticity, contributing to age-related deficits in sleep and temperature regulation, based largely on experimental animal studies and mechanistic neuroscience research [9,41]. Experimental animal studies indicate that warm-sensitive neurons within the preoptic hypothalamus coordinate heat-defence responses by simultaneously reducing heat production and enhancing heat dissipation via anatomically distinct pathways, suggesting that aging-related alterations in these neuronal populations may impair integrated thermoregulatory responses, as demonstrated in experimental rodent and circuit-level studies [42].

Aging impairs thermoregulatory function through combined central and peripheral mechanisms. Reduced thermal sensitivity and neurotransmitter imbalance in the preoptic area (POA) disrupt integration of thermoeffector responses, while peripheral deficits in vasodilation and sweating limit heat dissipation. These changes promote heat storage and nocturnal hyperthermia. Because thermoregulation and sleep are tightly coupled, impaired heat loss delays sleep initiation and destabilizes sleep architecture, resulting in fragmentation, reduced slow-wave sleep, and increased cardiometabolic risk. Hormonal changes, particularly reduced estrogen, further exacerbate thermoregulatory instability.

Thermoregulatory and sleep processes are tightly coupled through shared hypothalamic integration within the preoptic-anterior hypothalamus, which processes thermal, environmental, and vigilance-related inputs to coordinate both sleep–wake states and body temperature regulation [43]. Increased peripheral heat loss, particularly via distal skin warming, facilitates sleep initiation and consolidation, whereas impaired heat dissipation in aging may delay sleep onset and destabilize sleep architecture Experimental human sleep studies indicate that even subtle increases in skin temperature (~0.4 °C), without altering core temperature, significantly enhance slow-wave sleep and reduce nocturnal awakenings, especially in older adults, likely through modulation of sleep-regulatory neural circuits and cortical oscillatory activity [44].

Hormonal changes, particularly declining estrogen during menopause, can exacerbate thermoregulatory instability, manifesting as hot flashes and night sweats, which further disrupt nocturnal cooling and sleep. Clinical and translational evidence suggests that estrogen-related alterations in hypothalamic thermosensitive circuits and autonomic regulation contribute to these sleep-associated thermoregulatory disturbances [45,46].

Sex-related differences in thermoregulatory aging represent a clinically important but underexplored dimension of heat-induced sleep vulnerability. Menopause-associated estrogen withdrawal profoundly alters hypothalamic thermosensitivity, lowering the thermoneutral zone and reducing the threshold for vasodilatory and sudomotor activation, thereby increasing susceptibility to nocturnal hyperthermia and vasomotor symptoms such as hot flashes and night sweats [45,46]. These episodes directly fragment sleep architecture, reduce slow-wave sleep continuity, and impair the circadian temperature decline required for sleep initiation [43]. In older men, aging is associated with reduced peripheral vasodilatory capacity and impaired autonomic thermoregulatory responses, although these changes generally appear less pronounced than the abrupt thermoregulatory alterations accompanying the menopausal transition [8,38,45]. Furthermore, sex differences extend beyond hormonal modulation: women tend to exhibit lower absolute sweat rates and earlier onset of cutaneous vasodilation relative to core temperature thresholds, whereas men demonstrate higher sweat output but greater cardiovascular strain during heat exposure [36,37]. These divergent thermoregulatory phenotypes imply that heat-induced sleep disruption in aging populations is not homogeneous but is substantially shaped by biological sex, and that sex-stratified analyses are necessary to accurately characterize vulnerability and inform intervention design (Table 3).

These aging-related thermoregulatory deficits are discussed here in terms of their physiological basis; their specific interaction with heat exposure and sleep disruption is examined in detail in Section 5.

## 5. Heat and Aging: Converging Vulnerabilities for Sleep Disruption

This review integrates evidence from epidemiological, clinical, and experimental studies to explore the relationship between heat exposure, thermoregulation, and sleep disruption in aging populations. To address conceptual complexity, this section adopts a hierarchical framework in which thermoregulatory and circadian disruption are considered primary drivers of sleep disturbance under heat exposure. Downstream metabolic, immune, cardiovascular, and neurobiological changes are interpreted as secondary consequences of these primary sleep–temperature interactions.

Importantly, the strength of evidence varies across levels of biological organization. Human studies primarily demonstrate associations between environmental heat and sleep outcomes, whereas mechanistic insights into pathways such as AQP4-mediated glymphatic clearance, AMPK–mTOR–SIRT1 signaling, and clock gene regulation are largely derived from animal and in vitro models.

Therefore, the pathways described in this review should be interpreted as a hierarchical framework, distinguishing established human physiological responses from experimentally derived mechanistic hypotheses.

**Figure 2:**
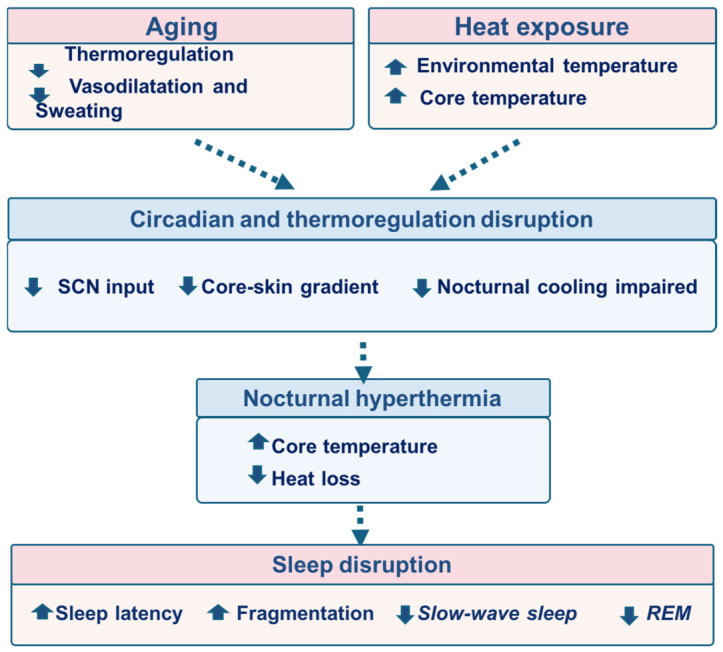
Converging effects of aging and heat exposure on sleep and systemic physiology.

Available human and experimental evidence suggests that aging and heat exposure may interact to increase vulnerability in older adults, particularly through their combined effects on sleep physiology. Building on the herein-previously described (see Section 4.2) aging-related thermoregulatory decline, heat exposure further elevates nocturnal core temperature, disrupting the normal decrease in core body temperature that facilitates sleep onset, thereby exacerbating sleep fragmentation and impairing slow-wave and REM sleep [47,48]. Large-scale human epidemiological studies further demonstrate that elevated nighttime temperatures significantly reduce sleep duration and increase sleep insufficiency, with older adults exhibiting greater vulnerability to heat-related sleep loss [49], consistent with findings from population-based vulnerability studies highlighting age-, sex-, and socioeconomic-status-dependent risk gradients [50]. Importantly, large-scale population analyses further show that the magnitude of sleep loss induced by ambient heat is not uniform globally, but varies substantially across climate zones and socioeconomic contexts, with stronger effects observed in warmer regions and populations with limited adaptive capacity [49,51]. For example, large-scale analyses have shown that each 1 °C increase in nighttime temperature is associated with measurable reductions in sleep duration, with effect sizes varying by climate zone and socioeconomic context. Heat-associated physiological stress has been associated with longer sleep latency, increased nocturnal awakenings, and potential disruption of melatonin-associated cooling processes, amplifying the fragmentation characteristic of late-life sleep (as illustrated in Figure 2).

Importantly, polysomnography-based studies provide objective evidence that heat exposure alters sleep architecture, including reductions in sleep efficiency and slow-wave sleep, and increased nocturnal awakenings, complementing epidemiological findings with direct physiological sleep measurements. Quantitative comparisons across human studies indicate that each 1 °C increase in nighttime ambient temperature is associated with reductions in sleep duration of approximately 10–30 min in population-based cohorts [2], while polysomnography-based studies demonstrate measurable decreases in sleep efficiency and slow-wave sleep during heat exposure, particularly in older adults compared to younger populations [52,53]. Controlled experimental studies show that adaptive thermal regulation can improve objective sleep quality, including sleep efficiency and stabilization of sleep stages [52], supporting translational evidence beyond observational epidemiology. However, experimental and field-based evidence also indicates that heat-related alterations in sleep and cognitive performance are strongly context-dependent, with pronounced impairments observed during heat waves and in non-air-conditioned environments, highlighting the modifying role of built infrastructure and environmental exposure conditions [54].

Aging-related thermoregulatory decline and environmental heat exposure act synergistically to disrupt circadian and thermal homeostasis. Reduced suprachiasmatic nucleus (SCN) signaling, diminished peripheral vasodilation, and decreased amplitude of core body temperature rhythms impair nocturnal heat dissipation, resulting in elevated nighttime core temperature. This disrupts sleep initiation and maintenance, leading to increased sleep latency, fragmentation, and reductions in slow-wave and rapid eye movement (REM) sleep. These disturbances propagate across multiple physiological systems, contributing to impaired glymphatic clearance in the brain, metabolic dysregulation via hypothalamic–pituitary–adrenal (HPA) axis activation, cardiovascular strain through sympathetic overactivity and endothelial dysfunction, and immune imbalance characterized by reduced adaptive responses and increased inflammation.

Human studies in older adults show that aging-related circadian and sleep disturbances disrupt the temporal coordination between thermoregulation and sleep architecture. Van Someren el al. (2000) indicated that aging is associated with decreased input to the SCN which accelerates deactivation of neurons responsible for generating 24 h rhythms [55]. These changes are thought to contribute to disrupted sleep–wake cycles, though they can be partially reversed by appropriate environmental or behavioral stimuli that reactivate SCN neurons. Extending this framework, Van Someren et al. (2002) emphasized the functional significance of circadian temperature rhythms: peripheral vasodilatation during the evening actively redistributes heat from the core to the skin, facilitating the nocturnal decline in core temperature necessary for sleep onset [56]. With aging, both rhythm amplitude and vasodilatory responsiveness decrease, weakening the core–skin temperature gradient and impairing physiological preparation for sleep, thereby increasing vulnerability to environmental stressors such as elevated nighttime temperatures. Moreover, it has been proposed that circadian fluctuations in core and skin temperature provide an additional signaling pathway for sleep–wake modulation, and that appropriately timed interventions (e.g., passive heating, bright light, melatonin) can partially restore sleep quality in older adults [55]. Changes in skin temperature with age directly affect sleep propensity, as elderly individuals show attenuated heat-loss activation prior to sleep onset, which can slow sleep initiation.

Human thermoregulatory adaptability remains present in older adults, although attenuated. Short-term passive heat acclimation studies demonstrate partial preservation of adaptive responses in both young and older adults, though magnitude and efficiency of adaptation are reduced with aging [57]. Similarly, human exercise-heat physiology studies confirm that older adults retain adaptive capacity but exhibit diminished thermoregulatory flexibility compared with younger populations [53,58,59].

Furthermore, inter-population variability is a key determinant of heat–sleep vulnerability. Importantly, this heterogeneity reflects not only biological aging processes but is also strongly shaped by climatic and environmental modifiers, including regional differences in ambient temperature profiles, humidity, urban heat island intensity, and access to adaptive infrastructure such as air conditioning, which collectively shape thermoregulatory efficiency and sleep outcomes across populations. Effect sizes of heat-related sleep disruption are consistently larger in warmer climatic zones and urban environments characterized by higher nocturnal heat retention, whereas cooler regions exhibit attenuated associations, highlighting clear geographic heterogeneity in vulnerability profiles [49,51]. These environmental modifiers may influence thermoregulatory efficiency by altering heat dissipation capacity and nocturnal cooling gradients. Systematic reviews and global evidence syntheses confirm that such heterogeneity in heat–sleep associations is consistent across different geographic regions and climatic conditions, demonstrating that ambient heat–sleep relationships are modified by climate zone, adaptation capacity, and infrastructural access, rather than representing a uniform physiological response, with consistent effect modification observed across climatic regions in population-based studies [2,4]. Epidemiological evidence shows that heat-related sleep disruption is strongly modulated by sex, socioeconomic status, and race/ethnicity [50]. Clinical sleep studies further demonstrate heterogeneity in objective sleep quality across diverse older adult populations, indicating that structural and demographic factors interact with biological aging processes [60]. Sex-related differences in thermoregulation across the lifespan further support variability in heat resilience [61].

At the population level, epidemiological evidence also shows that heat exposure is associated with both reduced sleep duration and impaired sleep health outcomes across geographically and climatically diverse regions, indicating strong environmental modulation of sleep vulnerability [2].

Foot warming interventions, such as warm footbaths or heated socks, have been reported to facilitate sleep onset in adults but are less effective in older adults, particularly those with insomnia [44]. The circadian rhythm of core temperature undergoes aging-dependent alterations, influenced by both homeostatic and circadian control mechanisms, with consequences for physiological functions and overall health [55]. These alterations interact with other circadian functions, contributing to the internal temporal order necessary for optimal functioning and highlighting potential targets for interventions in the elderly.

### 5.1. Molecular and Neurobiological Mechanisms

Evidence from animal studies suggests that sleep disruption may alter aquaporin-4 (AQP4) polarization and impair glymphatic clearance, thereby reducing the removal of neurotoxic metabolites such as β-amyloid and tau proteins. Human evidence, however, remains indirect and is primarily based on imaging and observational sleep studies rather than direct mechanistic confirmation.

**Figure 3:**
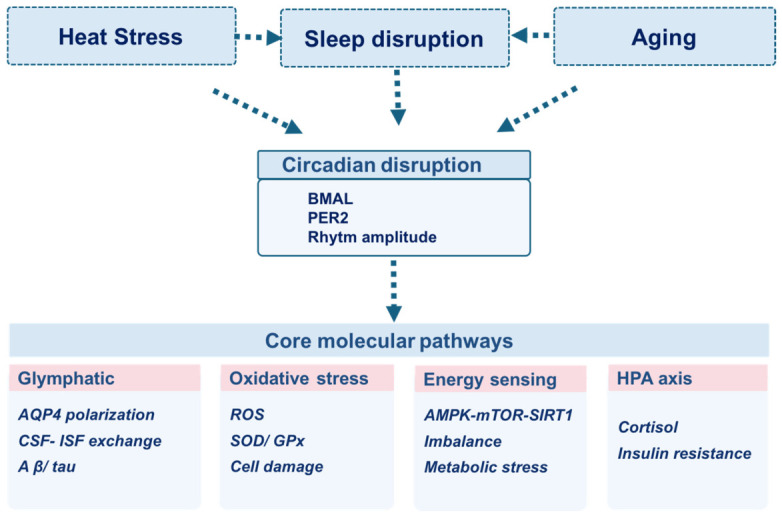
Mechanisms of Heat, Aging and Sleep disruption converging vulnerability.

At the cellular level, experimental evidence suggests that nocturnal hyperthermia may impair glymphatic clearance by disrupting the perivascular polarization of aquaporin 4 (AQP4) in astrocytic endfeet, thereby limiting cerebrospinal fluid–interstitial fluid exchange and reducing the removal of neurotoxic proteins such as β amyloid and tau (as illustrated in Figure 3) [62]. This mechanism is primarily supported by experimental animal and rodent models, which form the basis of current mechanistic understanding of glymphatic function in vivo. Foundational evidence from animal studies has established the glymphatic system as a sleep-dependent clearance pathway facilitating cerebrospinal fluid–interstitial fluid exchange and the removal of metabolic waste products from the brain [63,64].

Moreover, experimental and translational studies by Frey et al. (2025) and Salman et al. (2021) suggest that diurnal variations in AQP4 expression may be partly driven by circadian regulation, which modulates glymphatic flow and, consequently, indicates that circadian misalignment can further impair clearance mechanisms [65,66]. These observations are largely derived from experimental and translational neuroscience studies, including animal models and human expression/circadian association data, rather than direct causal human intervention studies. In addition, preclinical and translational evidence summarized by Costea et al. (2025) suggests an association between AQP4 dysfunction and accumulation of neurotoxic proteins, thereby contributing to neurodegenerative vulnerability [67]. However, this evidence is primarily based on experimental and preclinical findings.

Most depicted mechanistic links are based on preclinical evidence; direct human validation remains limited. At the molecular level, heat stress and sleep disruption, compounded by aging, induce circadian misalignment and dysregulation of clock genes (e.g., BMAL1, PER2), leading to widespread cellular dysfunction. Impaired polarization of aquaporin-4 (AQP4) disrupts glymphatic clearance and promotes accumulation of neurotoxic proteins such as β-amyloid and tau. Concurrently, mitochondrial dysfunction increases reactive oxygen species (ROS) production while reducing antioxidant defenses (e.g., superoxide dismutase and glutathione peroxidase), resulting in oxidative damage. Energy-sensing pathways, including AMPK–mTOR–SIRT1 signaling, become dysregulated, further compromising metabolic flexibility. Activation of the HPA axis elevates cortisol levels, promoting insulin resistance and metabolic imbalance. Together, these converging mechanisms drive neurodegenerative, metabolic, cardiovascular, and immunological vulnerability in aging populations exposed to heat stress. Importantly, most mechanistic links in this figure are derived from animal experiments and cellular models, with limited direct validation in aging human populations exposed to heat stress.

Furthermore, sleep fragmentation under thermal stress elevates mitochondrial reactive oxygen species (ROS) and downregulates antioxidant defenses, including superoxide dismutase (SOD) and glutathione peroxidase (GPx), which promotes oxidative damage to neuronal membranes and synaptic proteins, as shown in experimental animal studies [68,69]. In line with this, Grubač et al. (2021) demonstrated in rat model that the anxiogenic and cognitive impairments induced by sleep fragmentation are duration-dependent and mediated through oxidative stress, thereby linking oxidative injury directly to functional neural deficits [70].

Additionally, concurrent circadian misalignment alters clock gene expression (BMAL1, PER2), destabilizing transcriptional control of metabolic and inflammatory pathways [71,72]. Evidence linking circadian clock gene dysregulation to sleep and metabolic disturbances includes both human observational studies and mechanistic findings from experimental animal models; however, causal pathway integration under heat stress conditions remains incompletely established in humans. As a result, Irwin (2022) reports that these changes compromise neural plasticity, cognitive resilience, and the temporal regulation of protein clearance, which further increases vulnerability to neurodegenerative processes [73]. Therefore, the combined effects of heat stress, sleep disruption, oxidative imbalance, and circadian misalignment converge on AQP4 dysfunction and impaired glymphatic clearance, ultimately potentiating β amyloid and tau accumulation in the brain [66,67]. However, much of this evidence derives from animal models, and further human studies are needed to fully elucidate the extent to which glymphatic dysfunction contributes to neurodegenerative processes under conditions of heat stress and sleep disruption.

### 5.2. Contradictions in the Thermoregulatory Modulation of REM and NREM Sleep

The relationship between thermoregulatory processes and sleep architecture is well established at a general level. However, the specific effects of environmental temperature on REM and NREM sleep remain inconsistent across studies. Evidence derived from human polysomnographic studies, together with mechanistic findings from experimental and animal models, collectively supports the notion that sleep is tightly coupled to thermoregulatory function. Nevertheless, the direction and magnitude of REM/NREM modulation vary depending on methodological approach, population characteristics, and thermal exposure paradigms.

During NREM sleep, thermoregulatory heat loss mechanisms are functionally enhanced, whereas REM sleep is characterized by a relative attenuation of autonomic thermoregulatory control [74]. Within this framework, sleep initiation and maintenance are facilitated by a decline in core body temperature, supported by heat dissipation processes such as peripheral vasodilation and conductive heat loss [43]. These concepts are primarily based on physiological human sleep studies and integrative experimental sleep research. Czeisler and Gooley further emphasize that circadian regulation interacts with these thermoregulatory processes, such that sleep stage distribution is influenced by both circadian phase and homeostatic temperature regulation rather than temperature alone [75].

Experimental and polysomnographic studies provide converging but not fully consistent evidence regarding how ambient temperature alters sleep architecture. Enhanced conductive body heat loss has been shown to increase slow-wave sleep and improve cardiovascular stability during sleep [76], suggesting a robust link between facilitated heat dissipation and deeper NREM sleep expression. Similarly, adaptive thermal regulation interventions improve overall sleep efficiency and stabilize sleep architecture, including both REM and NREM sleep parameters [52]. These findings support a beneficial role of optimized thermal environments on sleep continuity and NREM depth.

However, REM sleep responses to thermal manipulation appear more heterogeneous. Okamoto-Mizuno and Mizuno (2012) reported that elevated ambient temperatures are consistently associated with increased sleep fragmentation and reduced sleep quality, but effects on REM sleep duration and proportion are less consistent across studies [48]. This variability suggests that REM sleep may be less directly governed by peripheral thermoregulatory mechanisms and more influenced by central regulatory processes.

Mechanistic insights further support this interpretation. Experimental neurobiological studies in animal models demonstrated that REM sleep is dynamically modulated by ambient temperature through pathways involving the melanin-concentrating hormone (MCH) system. Komagata et al. (2019) demonstrated that REM sleep is dynamically modulated by ambient temperature through neurobiological pathways involving the melanin-concentrating hormone (MCH) system, indicating that REM regulation is not solely a passive consequence of thermoregulatory load but involves specific hypothalamic circuitry responsive to thermal environment [77]. This provides a potential explanation for the inconsistent REM findings across human studies, as modulation may depend on the interaction between environmental temperature and central neuropeptidergic regulation rather than thermal load alone.

Integrative reviews of sleep thermoregulation further highlight that the coupling between temperature and sleep architecture is bidirectional and state dependent. Kräuchi and Deboer (2010) propose that thermoregulatory adjustments primarily facilitate NREM sleep initiation and maintenance, whereas REM sleep is maintained under a different regulatory regime with reduced thermoregulatory feedback sensitivity [43]. These integrative models combine evidence from human studies with mechanistic experimental evidences. Consequently, environmental heat exposure may exert stronger and more consistent effects on NREM-related processes (e.g., slow-wave sleep intensity and sleep continuity) than on REM sleep architecture.

These findings suggest that the modulation of REM and NREM sleep by thermoregulatory mechanisms is not uniform but context dependent. NREM sleep appears more tightly linked to peripheral heat loss and thermoregulatory efficiency, whereas REM sleep is influenced by a combination of circadian timing, central neurochemical regulation, and indirect thermal effects. While alterations in sleep architecture under heat exposure are supported by human sleep studies, several proposed mechanistic pathways underlying REM regulation are primarily derived from experimental and animal models and should therefore be interpreted cautiously in the context of human physiology.

### 5.3. HPA Activation and Metabolic Consequences

Heat stress, when coupled with sleep disruption, has been associated with activation of the HPA axis, resulting in elevated circulating cortisol levels. This hypercortisolemia may impair insulin receptor signaling by reducing IRS-1 and Akt phosphorylation, thereby promoting systemic insulin resistance [78,79], as shown in a randomized crossover study involving 14 subjects. Furthermore, Gulyaeva et al. (2022) discussed that chronic HPA axis activation modulates hippocampal glucocorticoid receptors (GR/NR3C1), consequently altering central regulation of glucose metabolism and energy homeostasis based on evidence from animal and human studies [80]. Concomitantly, dysregulation of appetite-regulating hormones, characterized by diminished leptin and elevated ghrelin levels, favors a positive energy balance, thereby exacerbating metabolic dysfunction. Associations between sleep curtailment, leptin dysregulation, obesity, and diabetes are supported mainly by human clinical and observational evidence, although mechanistic endocrine pathways remain incompletely understood [81]. In addition, heat-induced dehydration has been linked to impaired renal glucose handling and elevated circulating pro-inflammatory cytokines, including IL-6 and TNF-α, which collectively impair pancreatic β-cell function and disrupt systemic metabolic homeostasis [82].

Kuppuswami and Senthilkumar (2023) emphasize in their review of current literature, predominantly experimental cellular and animal studies, the pivotal role of heat shock proteins (HSPs) as central modulators of intracellular energy homeostasis under conditions of nutri-stress and mitochondrial dysfunction [83]. These molecular chaperones safeguard critical nodes within insulin signaling pathways, and their functional impairment exacerbates insulin resistance by attenuating cytoprotective mechanisms, thereby propagating cellular metabolic stress. Consequently, mitochondrial dysfunction induced by thermal or metabolic stress further has been linked to impaired insulin signaling, establishing a direct link between cellular energy imbalance and systemic glucose dysregulation. Collectively, the interplay of HPA axis hyperactivity, hormonal perturbations, dehydration, systemic inflammation, and HSP impairment synergistically amplifies insulin resistance and metabolic vulnerability. However, these integrated metabolic pathways should be interpreted as a translational and mechanistic framework synthesized from human observational findings together with experimental animal and molecular studies [83,84].

### 5.4. Cardiovascular and Autonomic Nervous System

During heat exposure, sympathetic nervous system activation increases, resulting in elevated catecholamine release (e.g., norepinephrine and epinephrine), which raises heart rate, blood pressure, and cardiovascular variability as demonstrated in both human and experimental heat-stress studies [85,86]. This increased sympathetic activity can overload the cardiovascular system, particularly in older adults or individuals with pre-existing cardiac conditions. Simultaneously, endothelial function may be impaired, largely due to reduced nitric oxide (NO) bioavailability and increased oxidative stress, which compromises vasodilation and overall vascular homeostasis based on clinical, translational, and experimental cardiovascular evidence [87].

Chen et al. demonstrated that age-related circadian dysregulation of neuroimmune cells, including astrocytes and microglia, elevates basal inflammation and disrupts rhythmic immune signaling primarily in experimental and translational aging models [88]. These changes can further exacerbate endothelial dysfunction and cardiovascular vulnerability in older adults, linking circadian and immune dysregulation to systemic cardiovascular risk. Jointly, these physiological responses exacerbate age-related cardiovascular vulnerabilities, predisposing individuals to arrhythmias, hypertension, myocardial ischemia, and cerebrovascular events according to human clinical and epidemiological evidence, supported by mechanistic experimental studies [14,87]. Moreover, repeated or prolonged heat exposure may amplify systemic inflammation and endothelial dysfunction, further increasing the risk of adverse cardiovascular outcomes.

### 5.5. Immunological Implications

Sleep disruption under heat stress has been associated with impaired immune function, including suppression of adaptive immunity and promoting inflammatory signaling. Normal sleep supports T cell activation and IL-2 production, which are essential for clonal expansion and effective adaptive responses, whereas sleep loss has been linked to reductions in these processes and disruption of cytokine balance based primarily on human experimental sleep deprivation studies supported by animal and mechanistic evidence [89,90]. Alongside, sleep disturbance activates NF-κB–mediated inflammatory pathways, leading to chronic low-grade inflammation and increased proinflammatory cytokine expression as demonstrated in both human studies and experimental mechanistic model [73]. Heat-induced sleep disruption further exacerbates these effects by interfering with thermoregulatory processes and sleep–immune coordination, resulting in diminished T cell responses, impaired vaccine efficacy, increased susceptibility to infection, and accelerated inflammaging in older adults based on converging evidence from human epidemiological studies with supporting experimental and translational findings [89,90].

### 5.6. Integrated Sleep–Thermoregulation Vulnerability

The evidence discussed in this section derives from a combination of experimental animal studies, human observational research, and mechanistic models. Therefore, causal interpretations in humans should be made taking this into account.

Impaired thermoregulation in older adults reduces the capacity to dissipate heat, whereas aging-associated sleep deterioration diminishes restorative processes, thereby potentially contributing to a mutually reinforcing cycle. Beyond delaying sleep onset and reducing slow-wave and REM sleep, sleep disruption has been shown to impair cellular maintenance pathways, including autophagy-mediated proteostasis, which are critical for neuronal health, as demonstrated in experimental models primarily involving preclinical animal systems such as Drosophila and rodent model [91].

Consistent with this, both animal and human studies indicate that sleep deprivation and aging are metabolically linked across tissues, underscoring conserved mechanisms that influence energy homeostasis and thermoregulatory efficiency based on a combination of human observational studies and experimental animal models [92,93]. However, human evidence in this context is largely observational and associative.

Energy sensing pathways, particularly the AMPK–mTOR–SIRT1 axis, are vulnerable to heat stress and sleep fragmentation. However, evidence linking this pathway to thermoregulatory responses in humans exposed to heat stress is still limited and largely indirect based primarily on experimental and cellular models with limited human mechanistic confirmation [93]. AMPK functions as a central energy sensor coordinating cellular energy balance across tissues and its role in systemic thermoregulatory adaptation in humans remains largely inferential. Mechanistic evidence on AMPK-related thermogenic and metabolic regulation is predominantly based on experimental animal studies and cellular models rather than controlled human interventions. The translation of these mechanistic findings to human thermoregulatory responses is limited by interspecies differences in energy metabolism, thermogenic adipose tissue activity, and hypothalamic control of energy balance. Zheng et al., demonstrated in mice that sleep deprivation stimulates adaptive thermogenesis via AMPK activation, increasing the expression of thermogenic genes in brown and white adipose tissue, which directly links sleep loss to altered thermoregulatory energy responses [94]. Rendine et al. reported that chronic sleep restriction in male mice upregulates SIRT1 and associated metabolic gene expression in white adipose tissue, suggesting alterations in energy metabolism that could influence thermoregulatory responses [95]. Chang et al., showed that melatonin administration preserves SIRT1 expression in the hippocampus of sleep-deprived rats, suggesting a protective mechanism mitigating sleep loss–induced metabolic and thermoregulatory stress [96]. These findings collectively suggest mechanistic involvement of AMPK–SIRT1 signaling in cellular energy utilization and stress adaptation pathways, particularly under conditions of sleep loss.

Therefore, aging-related functional decline, environmental heat exposure, and sleep loss converge to produce an integrated pathway linking environmental stressors to neurodegenerative, metabolic, cardiovascular, and immune dysfunction [97]. A critical limitation of the mechanistic evidence synthesized in this review is the substantial reliance on rodent and other animal models, which may introduce a translational gap when applying these findings to aging human populations. While experimental animal studies have provided foundational insights into AQP4-mediated glymphatic clearance, AMPK–SIRT1 signaling, and clock gene regulation under heat stress, direct human validation of these pathways remains limited. Importantly, sleep and thermoregulation are tightly coupled to ambient temperature dynamics in humans, and heat exposure has been shown to exert profound effects on sleep architecture and neurophysiological stability [84]. Humans also possess a uniquely powerful behavioral thermoregulatory capacity that animal models cannot replicate: the ability to actively modify their thermal environment through actions such as adjusting air conditioning, changing clothing, altering sleep schedules, or relocating to cooler spaces. This behavioral flexibility fundamentally shapes the real-world impact of heat exposure on sleep and thermoregulation in older adults, and its absence in rodent paradigms limits the direct transposition of preclinical findings. Furthermore, interspecies differences in autonomic cardiovascular responses and heat stress adaptation introduce additional layers of biological non-equivalence [87]. Sleep loss and heat exposure are also closely linked to systemic metabolic and inflammatory dysregulation across tissues, further complicating direct mechanistic extrapolation from animal models [92]. Importantly, translational evidence from controlled heat-wave simulation studies in older adults demonstrates that environmental cooling interventions can significantly mitigate physiological strain, underscoring the clinical relevance of real-world environmental modulation beyond molecular pathways [97]. Collectively, these considerations underscore the necessity of controlled human intervention studies and longitudinal cohort designs specifically targeting heat-exposed older populations before mechanistic conclusions derived from animal models can be confidently translated into clinical or public health recommendations.

Accordingly, interventions that restore circadian rhythm integrity, enhance cellular energy and redox homeostasis, and support thermoregulatory efficiency may attenuate the compounded risks associated with heat stress and sleep deterioration in aging populations.

To improve conceptual clarity, the complex interactions described in this section can be summarized in a simplified model. Heat exposure interacts with age-related thermoregulatory and circadian decline, leading to impaired nocturnal cooling and sleep disruption as primary events. These sleep disturbances then act as central mediators, propagating downstream effects on metabolic regulation, autonomic function, immune responses, and neurobiological processes. This hierarchical model emphasizes sleep and thermoregulatory dysfunction as the core integrating pathway linking environmental heat to systemic aging-related vulnerability.

Table 4 summarizes the systemic effects of heat waves and sleep disruption on aging populations, integrating molecular, metabolic, cardiovascular, immune, and sleep-related vulnerabilities.

This table distinguishes primary physiological drivers (thermoregulatory and sleep disruption) from downstream systemic consequences, providing a structured hierarchy of heat-related aging vulnerability.

### 5.7. Implications for Public Health and Intervention

Environmental cooling strategies, such as access to cooling centers or air-conditioned environments, have been shown in laboratory-based heat wave simulation studies in older adults to reduce physiological heat strain and lower core body temperature during acute heat exposure [97]. Importantly, a large case-crossover study of nursing home residents in Ontario, Canada (2010–2023) found that access to air conditioning was associated with lower odds of mortality during extreme heat days compared with facilities without air conditioning, providing epidemiological support for cooling interventions in institutional settings [98].

Complementary observational evidence in community-dwelling older adults indicates that higher nighttime indoor temperatures are associated with reduced sleep duration and impaired sleep continuity, with sleep outcomes being most favorable within moderate thermal ranges and deteriorating under higher heat exposure conditions [99]. A systematic review further confirms that higher indoor temperatures are consistently associated with increased sleep disturbances in older adults, with stronger effects in socioeconomically disadvantaged and urban populations [100]. These epidemiological associations must be interpreted within a broader framework of environmental inequality, as socioeconomic and infrastructural factors function as critical effect modifiers rather than mere background variables. The urban heat island (UHI) effect substantially elevates nocturnal ambient temperatures in densely built environments, disproportionately affecting older adults residing in low-income urban neighborhoods where green space is limited, building insulation is poor, and access to air conditioning is constrained [98,99,100]. Consequently, the magnitude of heat-related sleep disruption observed in large-scale epidemiological studies is not solely a reflection of biological thermoregulatory vulnerability but is also shaped by differential exposure to thermal stress driven by housing quality, neighborhood infrastructure, and socioeconomic status [50,99]. Older adults with limited financial resources are less able to afford or operate cooling systems, increasing their cumulative nocturnal heat burden and compounding age-related thermoregulatory decline [39,53]. These structural inequalities mean that interventions targeting only physiological or behavioral adaptation, without addressing the environmental conditions in which older adults live, are likely to be insufficient. Public health strategies must therefore integrate housing policy, urban planning, and targeted cooling access programs alongside clinical and behavioral recommendations to meaningfully reduce heat-induced sleep disruption in the most vulnerable older populations, especially in urban environments [50,97,98]. However, most available evidence remains observational or short-term, limiting inference on long-term adaptation.

Importantly, large-scale wearable-based and population-level epidemiological studies provide quantitative evidence linking ambient heat exposure to reduced sleep duration across general adult populations. A global analysis of over 7 million sleep records shows that higher nighttime temperatures are associated with shorter sleep duration, an effect primarily driven by delayed sleep onset [51]. Similarly, a longitudinal cohort study using more than 12 million wearable sleep records found that a 10 °C increase in nighttime temperature anomaly is associated with a 2.63 min reduction in total sleep duration per night (95% CI: −2.51 to −2.75) [2]. Consistent with these findings, earlier survey-based epidemiological evidence demonstrates that increases in nighttime temperature are associated with a higher probability of reporting insufficient sleep [49].

Experimental field evidence further indicates that acute heat exposure can impair cognitive performance. In a controlled observational study, young adults living in non-air-conditioned buildings showed approximately 13% slower reaction times and reduced cognitive throughput during heat-wave conditions compared with those in air-conditioned environments [54]. Although this study assessed cognitive outcomes rather than sleep physiology, it supports the broader impact of heat exposure on neurobehavioral functioning.

Behavioral and circadian-aligned interventions are also critical, as heat stress disrupts sleep and further exacerbates physiological vulnerability in older adults [4]. Public health guidance, including that of the World Health Organization, emphasizes hydration, avoidance of peak heat exposure, access to cooling environments, and strengthened community support, particularly for socioeconomically disadvantaged older adults with limited access to cooling resources [101]. In addition, population-based studies indicate that ambient temperature fluctuations are associated with a broad range of adverse mental health outcomes, although these findings are primarily observational in nature [102]. These risks may be further exacerbated in urban environments due to the urban heat island effect, which increases nighttime temperatures and limits physiological recovery.

Melatonin has been reported to modulate circadian body temperature rhythm parameters in older adults. In a controlled clinical study, daily evening administration of 1.5 mg melatonin was associated with reduced inter-individual variability in circadian phase and increased rhythm stability, as assessed using cosinor analysis, suggesting improved synchronization of circadian timing processes in elderly individuals [103].

Evidence from randomized controlled trials indicates that melatonin provides modest improvements in sleep outcomes in older adults, including reductions in sleep-onset latency and small increases in total sleep time. Prolonged-release formulations have been evaluated in placebo-controlled trials with follow-up periods extending up to several months, demonstrating sustained but modest efficacy without evidence of tolerance [104].

Clinical guidance generally suggests that low-dose melatonin (typically 0.5–2 mg, prolonged-release formulation) administered 1–2 h before bedtime may be appropriate in older adults. Clinical recommendations emphasize cautious use due to inter-individual variability in response and limited dose–response evidence [105,106].

Meta-analytic evidence in older and cognitively impaired populations suggests that melatonin may improve sleep duration and related outcomes; however, effect sizes are generally small to moderate and heterogeneity across studies is substantial [107]. Earlier meta-analyses similarly report modest improvements in sleep latency, total sleep time, and subjective sleep quality in primary sleep disorders [108].

More recent systematic reviews focusing on older adults with chronic insomnia (mean age ≥ 55 years) report improvements in sleep latency, total sleep time, and sleep quality with melatonin and/or melatonin receptor agonists compared with placebo, with generally favorable safety profiles, although methodological quality and effect sizes vary across trials [109].

These findings are consistent with earlier foundational clinical work, including the study by Gubin et al. (2006), which provided early evidence suggesting that melatonin may modulate circadian temperature rhythm parameters in older adults; however, this study is limited by its relatively small sample size and remains part of the early evidence base [103]. Overall, current evidence is derived from heterogeneous clinical studies not specifically designed for heat-exposed older populations, and heat-exposure–specific randomized controlled trials remain limited.

While circadian-targeted interventions such as timed light exposure, melatonin administration, and activity scheduling have been proposed to support circadian alignment in older populations, the strength of evidence for their effectiveness varies, and causal effects in heat-stressed aging populations remain insufficiently established.

Emerging technological and environmental adaptation strategies, including wearable cooling devices, predictive heat-risk modeling, and other experimental approaches to improving human thermoregulation, are being actively investigated as potential methods to reduce heat-related health risks in older adults [110]. However, most of these approaches remain at early stages of development, and robust evidence from large-scale clinical trials in older populations is still limited. Systematic and scoping reviews indicate that current heat adaptation strategies in older adults primarily focus on behavioral, environmental, and community-level interventions, with varying levels of effectiveness depending on context and implementation [111,112]. Recent evidence also suggests that community-based heat adaptation interventions may provide beneficial effects, although the strength of evidence remains heterogeneous and further controlled studies are needed [113]. Integrating environmental, behavioral, and technological strategies is increasingly emphasized in public health planning to reduce vulnerability to heat exposure in aging populations. In parallel, climate model projections based on epidemiological data indicate that temperature-related mortality risks are expected to increase under future warming scenarios, particularly among vulnerable groups such as older adults [114].

## 6. Conclusions

Heat waves, increasingly frequent and intense due to climate change, pose substantial health risks for older adults, whose aging-related declines in thermoregulatory capacity impair nocturnal cooling. Elevated nighttime temperatures disrupt sleep onset, slow-wave and REM sleep, and melatonin secretion, potentially contributing to impaired glymphatic clearance, oxidative stress, circadian misalignment, metabolic dysregulation, cardiovascular diseases, and immune system decline. Reduced POA neuron sensitivity altered GABA/glutamate balance, and PACAP/BDNF pathway dysregulation, further increase susceptibility to heat-induced sleep disruption. The convergence of aging, heat exposure, and sleep disruption may form a mutually reinforcing cycle, in which each component plausibly exacerbates the others. However, the precise directionality and magnitude of these interactions have not yet been firmly established in human studies. We propose that this dynamic amplifies neurodegenerative, metabolic, cardiovascular, and immune risks, highlighting the urgent need for interventions such as environmental cooling, circadian-aligned behavioral strategies, and targeted public health measures to protect older populations. Future research should prioritize heat-exposure-specific randomized controlled trials in older adults, longitudinal studies examining bidirectional sleep–thermoregulation interactions, and mechanistic human studies validating preclinical findings regarding AQP4, AMPK–SIRT1, and clock gene pathways.

## Figures and Tables

**Table 1:** Peripheral–Central Integration of Thermoregulation.

Step/Component	Description	References
Peripheral detection	Thermal information from the environment is sensed by thermoreceptors in skin, mucous membranes, and deep tissues. Warm- and cold-sensitive afferent neurons detect temperature changes.	[16,17]
Afferent transmission	Signals are carried via Aδ and C fibers to the dorsal horn of the spinal cord, then relayed through brainstem nuclei such as the lateral parabrachial nucleus.	[16,17]
Central integration (POA)	The preoptic area (POA) integrates peripheral signals with central temperature information to coordinate thermoregulatory responses.	[15]
Efferent responses: Heat	Sympathetic-mediated vasodilation, sweating, behavioral adaptations (e.g., seeking cooler environments).	[15]
Efferent responses: Cold	Shivering, brown adipose tissue activation, peripheral vasoconstriction to conserve heat.	[15]
Endocrine & metabolic modulation	Hormonal signals such as thyroid hormones and other metabolic mediators modify thermoregulatory responses during physiological stress by regulating heat production, energy balance, and interacting with neural and immune pathways.	[18]

Aδ: A-delta fibers; C: C fibers; POA: Preoptic area.

**Table 2:** Major Molecular and Neuronal Mechanisms Involved in Central Thermoregulation.

Component Type	Component/Channel/Neuron	Location/Circuit	Function/Role	Key References
Thermosensitive Ion Channels (TRP family)	TRPM2	Preoptic area (POA) neurons	Senses elevated temperatures; activates heat-loss mechanisms (vasodilation, behavioral cooling); dysfunction → impaired heat dissipation, hyperthermia	[24,25]
	TRPM3, TRPM4, TRPM5	POA neurons	Contribute to neuronal thermosensitivity; detect temperature changes	[23]
	TRPV1	POA neurons; also peripheral sensory neurons	Detects high temperatures; estrogen fluctuations modulate expression and thermal sensitivity; involved in hot flashes	[26]
Inhibitory neurons	GABAergic neurons	POA (VLPO, MnPO)	Suppress thermogenesis during heat exposure; promote NREM sleep by inhibiting wake-promoting regions; contribute to nocturnal decline in core temperature	[9]
Excitatory neurons	Glutamatergic neurons	POA	Modulate heat production and heat-loss pathways; destabilize NREM sleep and suppress REM sleep depending on environmental conditions	[27]
Synaptic temperature sensors	Unspecified synaptic mechanisms	POA	Detect local temperature changes and contribute to rapid heat-loss responses	[22]

GABA: Gamma-aminobutyric acid; MnPO: Median preoptic nucleus; NREM: Non-rapid eye movement; POA: Preoptic area; REM: Rapid eye movement; TRP: Transient receptor potential; TRPM2: Transient receptor potential melastatin 2; TRPM3: Transient receptor potential melastatin 3; TRPM4: Transient receptor potential melastatin 4; TRPM5: Transient receptor potential melastatin 5; TRPV1: Transient receptor potential vanilloid 1; VLPO: Ventrolateral preoptic nucleus.

**Table 3:** Age-Related Alterations in Thermoregulation and Their Potential Health Consequences.

Age-Related Change	Physiological/Molecular Effect	Clinical Consequence
Reduced sweating	Impaired heat dissipation	Increased hyperthermia risk
Decreased vasodilation	Reduced heat loss	Heat stroke susceptibility
Altered POA neuron sensitivity	Reduced GABA/glutamat balance PACAP & BDNF signaling decline	Nighttime hyperthermia, sleep fragmentation
Hormonal changes	Estrogen decline affects TRPV1 and thermosensitive channels	Hot flashes, sleep disturbances

BDNF: Brain-derived neurotrophic factor; GABA: Gamma-aminobutyric acid; PACAP: Pituitary adenylate cyclase-activating polypeptide; POA: Preoptic area; TRPV1: Transient receptor potential vanilloid 1.

**Table 4:** Heat Waves, Sleep Disruption, and Aging: Systemic Effects.

System/Level	Mechanism/Effect	Outcome/Clinical Relevance
Neurological/Molecular	Nocturnal hyperthermia → disrupted AQP4 polarization → impaired glymphatic clearance	Accumulation of β-amyloid and tau → synaptic dysfunction, increased risk of neurodegeneration
	Increased ROS, decreased SOD and GPx → oxidative stress	Neuronal membrane and synaptic protein damage → cognitive decline
	Circadian misalignment → altered BMAL1/PER2 expression	Impaired neural plasticity, disrupted metabolic and inflammatory regulation
Metabolic/Endocrine	HPA axis activation → elevated cortisol	Insulin resistance, impaired glucose tolerance, increased metabolic syndrome risk
	Leptin ↓, Ghrelin ↑; dehydration → IL-6, TNF-α ↑	Positive energy balance, β-cell dysfunction, systemic inflammation
Cardiovascular/Autonomic	Sympathetic overactivation → catecholamine surge; endothelial dysfunction → reduced NO	Hypertension, arrhythmias, myocardial ischemia, increased cerebrovascular risk
Immune	T-cell activity ↓; IL-2 ↓; NF-κB ↑	Impaired adaptive immunity, chronic inflammation, higher susceptibility to infection
Sleep & Thermoregulation	Reduced slow-wave (N3) and REM sleep; decreased melatonin	Sleep fragmentation, circadian disruption → impaired cognitive and metabolic resilience
Integrated Systemic Impact	Combination of molecular, metabolic, cardiovascular, immune, and sleep disturbances	Neurodegeneration, metabolic dysfunction, cardiovascular stress, inflammation → increased vulnerability in older adults

AQP4: Aquaporin-4; BMAL1: Brain and muscle ARNT-like 1; GPx: Glutathione peroxidase; HPA: Hypothalamic–pituitary–adrenal; IL-2: Interleukin-2; IL-6: Interleukin-6; NF-κB: Nuclear factor kappa B; NO: Nitric oxide; PER2: Period circadian regulator 2; REM: Rapid eye movement; ROS: Reactive oxygen species; SOD: Superoxide dismutase; TNF-α: Tumor necrosis factor alpha. ↓: decrease, →: influence and ↑: increased.

## Data Availability

No new data were created or analyzed in this study. Data sharing is not applicable to this article.
